# Elucidating the Effect of Temperature Stress on the Protein Content, Total Antioxidant Capacity, and Antioxidant Enzyme Activities in *Tetranychus urticae* (Acari: Tetranychidae)

**DOI:** 10.3390/insects14050429

**Published:** 2023-04-29

**Authors:** Peng-Cheng Nie, Ruo-Lan Yang, Jing-Jiang Zhou, Youssef Dewer, Su-Qin Shang

**Affiliations:** 1 Biocontrol Engineering Laboratory of Crop Diseases and Pests of Gansu Province, College of Plant Protection, Gansu Agricultural University, Lanzhou 730070, China; niepcgs@163.com (P.-C.N.); yangrlhb@163.com (R.-L.Y.); jjzhou@gsau.edu.cn (J.-J.Z.); 2 State Key Laboratory of Green Pesticide and Agricultural Bioengineering, Ministry of Education, Guizhou University, Guiyang 550025, China; 3 Phytotoxicity Research Department, Central Agricultural Pesticide Laboratory, Agricultural Research Center, 7 Nadi El-Seid Street, Giza 12618, Dokki, Egypt; dewer72@yahoo.com

**Keywords:** *Tetranychus urticae*, heat stress, protein content, antioxidant activity, total antioxidant capacity

## Abstract

**Simple Summary:**

The two-spotted spider mite *Tetranychus urticae* Koch is an extremely polyphagous agricultural pest which shows a great tolerance to high temperatures. High temperatures can influence the protein content and are often associated with the generation of reactive oxygen species (ROS), which cause oxidative damage. To prevent the damage by ROS, organisms have developed antioxidant defense mechanisms, such as antioxidant enzymes, which can eliminate ROS. Moreover, total antioxidant capacity is a resultant measure of the ability of all antioxidants present in an organism to counteract the oxidation of an indicator by an oxidant, or to reduce an indicator substance. In the present study, the effects of high temperatures on protein content, antioxidant enzyme activities, and T-AOC in *T. urticae* were investigated. The results showed that protein content, antioxidant enzyme activities, and T-AOC were significantly induced by high temperatures (*p* < 0.05), which indicates that antioxidant enzymes increase the resistance of *T. urticae* in a range of 36–42 °C. In summary, this study enriches the understanding of the physiological mechanisms of resistance of *T. urticae* to environmental high temperatures.

**Abstract:**

*Tetranychus urticae* Koch is a worldwide agricultural pest mite that feeds on more than 1100 kinds of crops. The mite has developed a high level of tolerance to high temperatures, but the physiological mechanism underlying the outstanding adaptability of this pest to high temperatures remains unclear. To clarify the physiological mechanisms of *T. urticae* in response to short-term heat stress, four temperatures (36, 39, 42, and 45 °C) and three short-term heat durations (2, 4, and 6 h) were conducted to test the effects on protein content, the activities of superoxide dismutase (SOD), peroxidase (POD), and catalase (CAT), and the total antioxidant capacity (T-AOC). The results showed that protein content, antioxidant enzyme activity, and T-AOC in *T. urticae* were significantly induced by heat stress. These results suggest that heat stress induces oxidative stress and that antioxidant enzymes play an important role in reducing oxidative damage in *T. urticae*. The data of this study will provide a basis for further research on the molecular mechanisms of thermostability and ecological adaptability of *T. urticae*.

## 1. Introduction

The two-spotted spider mite *Tetranychus urticae* represents one of the key pest mites around the world and has a high capacity to adapt to a wide range of hosts. It can feed on many economic crops and ornamental plants, which covers over 1275 plant species from 70 genera [1,2,3]. *T. urticae* uses its mouthparts to penetrate host cells and ingest cell contents that lead to a decreased transpiration and photosynthesis activity decrease and delayed development of plants [4,5]. Yield can be reduced from 60% to 80% and, in extreme cases, the plant dies [6]. *T. urticae* has been considered as the most serious tetranychid pest species in orchards and greenhouses up to now due to its high level of insecticide resistance and high-temperature tolerance [7].

For all insects and mites, temperature is one of the most important environmental factors affecting their growth, reproduction, distribution, abundance, and phenology, as well as inducing their physiological responses [8,9,10,11]. It is reported that temperature stress, including both high and low temperatures, could break the balance between the generation of reactive oxygen species (ROS) and antioxidant defenses [12]. ROS include the superoxide anion ($O_{2}^{-}$), hydrogen peroxide (H_2_O_2_), and hydroxyl radical (HO·), all of which have inherent chemical properties that confer reactivity to different biological targets [13,14]. In normal situations, the low levels of ROS are essential for the normal physiological signal of transduction pathways that regulate complex processes in cells [15]. However, if unbalanced by antioxidant defenses, surplus ROS can damage biological macromolecules (nucleic acids, proteins, and lipids) and lead to cell damage, mutation, and even death [16,17], thereby reducing insect/mite survival, hindering development and reproduction, and significantly inhibiting the populations [18,19,20,21].

In order to maintain homeostasis and prevent oxidative stress as well as damage by ROS, living organisms have evolved a complex defense system, including various non-enzymatic molecules and antioxidant enzymes [22,23]. Major antioxidant enzymes of mites include superoxide dismutase (SOD), peroxidase (POD), and catalase (CAT) [24,25]. During the enzymatic reactions, SOD removes $O_{2}^{-}$ through the process of dismutation to O_2_ and H_2_O_2_ (2$O_{2}^{-}$ + 2H^+^ → H_2_O_2_ + O_2_) to prevent cell damage [17,26,27,28], while CAT and POD break H_2_O_2_ into H_2_O and O_2_ [29]. However, CAT removes H_2_O_2_ only at high cellular concentrations and is inefficient at removing H_2_O_2_ at low concentrations [30,31,32]. Total antioxidant capacity (T-AOC) is a measure of the ability of an organism to combat oxidation using all antioxidants [33].

Some reports have confirmed that antioxidant enzyme activities were enhanced to scavenge ROS to protect insects and mites from heat stress. For example, *T. cinnabarinus* (Boisduval) and *Mononychellus mcgregori* (Flechtmann and Baker) can respond to ROS produced by heat stress through increasing protective enzyme activity [34,35]. In *T. urticae*, the fluctuation of temperature is reported to influence the growth, development, survivorship, reproduction, activity of the detoxification enzyme, and expression of heat-shock cognate 70 (Hsc70) [36]. Recently, Hernández-Rivera et al. [6] studied the temperature–mortality relationship for different developmental stages (eggs, larvae, protonymphs, deutonymphs, and adult females) of *T. urticae*, which showed the potential of using temperature as an alternative to acaricides.

However, the response of antioxidant enzymes in *T. urticae* exposed to heat stress has not been reported yet. Therefore, the present study aimed to investigate the changes in the protein content, antioxidant enzyme activities, and T-AOC in response to the heat stress in *T. urticae*. Further, it was of interest to identify oxidative stress and evaluate the potential physiological responses of *T. urticae* to various treatment durations. These findings can provide a reference for the ecological adaptability of *T. urticae* that is required for fully understanding the molecular mechanisms of thermostability of this pest.

## 2. Materials and Methods

### 2.1. Mite Colony and Short-Term Heat Exposure

A colony of the two-spotted spider mite was originally obtained from Xinglong Mountain, Gansu Province, China, in May 2012, and considered as a susceptible strain. This colony population was maintained in a temperature-controlled room located in Gansu Agricultural University, Lanzhou, China, on bean *Phaseolus vularis* (L.) (Fabaceae) leaves for more than 30 generations without exposure to insecticides. The rearing condition was 25 ± 1 °C, 60 ± 5% RH, and an L16:D8 photoperiod.

The heat treatment was provided with an artificial climate chamber (Shanhai Yuejin, model HQH-H300, China). The newly emerged female adults (<24 h) were exposed to each target temperature, including 36 °C, 39 °C, 42 °C, and 45 °C. The durations of each temperature were 2, 4, and 6 h. The control group was kept at room temperature (25 ± 1 °C). After the short-term heat exposure, 150 surviving female adults of *T. urticae* from each treatment were collected and immediately frozen in liquid nitrogen and stored at −80 °C for the measurement of the protein content, enzyme activity, and total antioxidant capacity. Each treatment was replicated three times.

### 2.2. Preparation of the Enzyme Solutions and Protein Content Determination

The preparation of assay samples was based on the methods described by Li et al. [37] and Lu et al. [35] with some minor changes. After the short-term heat stress, 150 surviving females from each treatment were placed into a 2 mL centrifugal tube. Later, an extract solution of 1.5 mL phosphate buffer (PBS, 0.05 M, pH 7.0) was added and rapidly triturated with the samples. The mixture was then centrifuged at 10,000 rpm for 15 min at 4 °C. The supernatant was collected and stored at −80 °C until used as the sample solution.

Protein concentration was determined according to the Bradford method with bovine serum albumin as the standard [38]. The protein content was used to quantify the activities of SOD, CAT, and POD and the T-AOC. The absorbance values were obtained using a full-wavelength spectrophotometer (ELX800UV, Bio-Tek Instruments Inc., Winooski, VT, USA).

### 2.3. Enzyme Activity Assays of T. urticae

The activities of superoxide dismutase (SOD), peroxidase (POD), and catalase (CAT) and total antioxidant capacity (T-AOC) were determined with commercially available assay kits (YX-C-A500, YX-C-A502, YX-C-A501, and YX-C-A504, Shanghai Preferred Biotechnology, Shanghai, China). In the negative control, we replaced the enzyme solution with the same volume of phosphate buffer (PBS, 0.05 M, pH 7.0).

The method described in the instruction from the assay kit was followed. SOD activity was measured at 560 nm by xanthine and xanthine oxidase systems. One unit of SOD activity was defined as the amount of enzyme required to cause 50% inhibition of the xanthine oxidase system reaction in 1 mL enzyme extract with 1 mg protein (U/mg protein). CAT activity was determined at 240 nm by measuring the decrease in H_2_O_2_ due to hydrogen peroxide decomposition. One unit of CAT activity was defined as the amount that decomposes 1 nmol of H_2_O_2_ per second per mg protein (U/mg protein). POD activity was determined at 470 nm by catalyzing the oxidation of a substrate in the presence of H_2_O_2_. A change in absorbance at 470 nm of 0.01 per minute in 1 ml solution was defined as an enzyme activity unit. T-AOC was determined at 593 nm with FeSO_4_ as the standard. One unit of T-AOC was defined as the amount necessary to increase the absorbance by 0.01 per min per mg protein. T-AOC was expressed as U/mg protein.

### 2.4. Statistical Analysis

The statistical analyses were carried out by using the general linear model procedure of SPSS 26.0 software (SPSS, Inc., Chicago, IL, USA). The one-way ANOVA (followed by Duncan’s new multiple range method, *p* < 0.05) was applied to test the differences among the protein content and enzyme activities. The relations between temperature and treatment duration were analyzed with a two-way analysis of variance (ANOVA) and followed by Tukey HSD post hoc tests (*p* < 0.05). Origin 2018 (OriginLab, Northampton, MA, USA) was used to conduct the standard curve of bovine serum albumin calculation and figures.

## 3. Results

### 3.1. Total Protein Content

**Figure 1:**
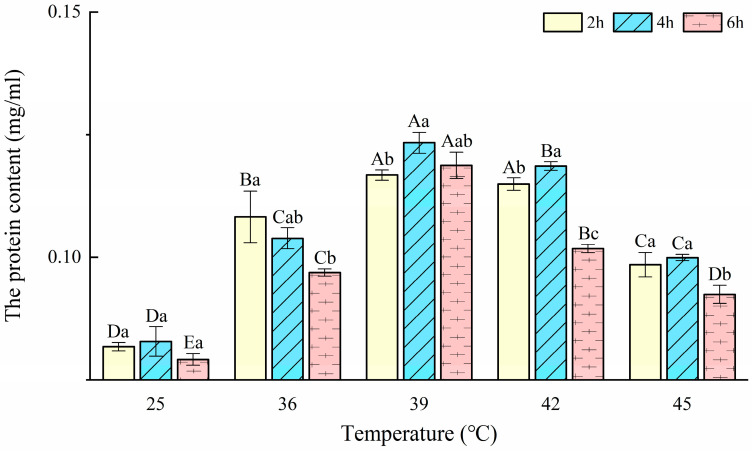
The protein content in *Tetranychus urticae* after short-term heat stress (mg/mL). Notes: Data are mean ± SE. Different capital letters indicate significant differences in the protective enzyme activities at the same exposure duration among different temperatures, while lowercase letters indicate significant differences at the same temperature among different exposure durations at 0.05 level by Duncan’s new multiple range test, the same as below.

The relation between the OD values (y) and the BSA concentrations (x) was y= 0.0038x + 0.03233 with an R of 0.99639. The changes in the protein content of *T. urticae* after the heat shock for different durations are presented in Figure 1. The protein contents of *T. urticae* were significantly affected by the treatment temperatures (F = 240.38; *p* < 0.001) and durations (F = 58.08; *p* < 0.001), and the interactions between these treatment factors were significant (F = 7.03; *p* < 0.001) according to the two-way ANOVA. After exposure to the heat stress for different durations, protein contents at 36, 39, 42, and 45 °C were all significantly higher than controls (F_2h_ = 54.86, *p*_2h_ < 0.001; F_4h_ = 84.14, *p*_4h_ < 0.001; F_6h_ = 170.33, *p*_6h_ < 0.001). The least protein content (0.0772 ± 0.0012 mg/mL) was formed at 25 °C-6 h and reached a maximum (0.1233 ± 0.0021 mg/mL) at 39 °C-4 h, which is higher than the control with 0.0928 ± 0.0030 mg/mL. The protein content of three durations presented a similar variation trend that increased at 25–39 °C and decreased at 39–45 °C.

### 3.2. Superoxide Dismutase (SOD) Activity

**Figure 2:**
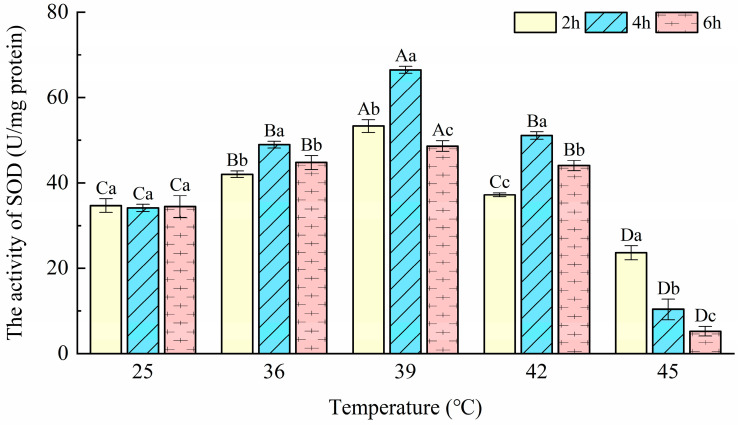
Effects of the activity of superoxide dismutase (SOD) in *Tetranychus urticae* under heat stress.

As shown in Figure 2, the SOD activity of *T. urticae* was significantly affected by the temperatures (F = 784.38; *p* < 0.001) and durations (F = 39.24; *p* < 0.001), and the interactions among these treatment factors were significant (F = 51.05; *p* < 0.001). After exposure to the heat stress for different durations, the activity of SOD in *T. urticae* reached the maximum (66.48 U/mg protein) at 39 °C-4 h, which is more than twice the control at 28.52 U/mg protein. The minimum (5.26 U/mg protein) appeared at 45 °C-6 h, which is nearly one-seventh of the control with 34.46 U/mg protein. The SOD activity showed the same trend: it first increased from 25 °C to 39 °C, and decreased from 39 °C to 45 °C. SOD activity after 2-, 4-, and 6-h treatments at 45 °C was significantly lower than that at 25 °C (F_2h_ = 141.80, *p*_2h_ <0.001; F_4h_ = 532.90, *p*_4h_ < 0.001; F_6h_ = 231.76, *p*_6h_ < 0.001).

### 3.3. Peroxidase (POD) Activity

**Figure 3:**
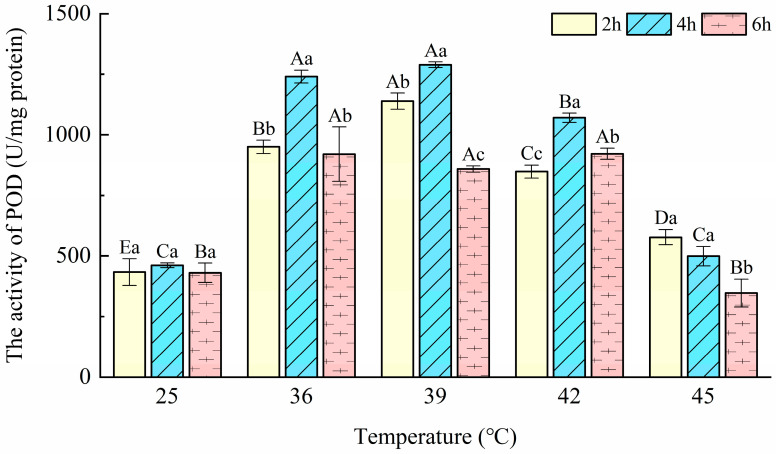
Effects of the activity of peroxidase (POD) in *Tetranychus urticae* under heat stress.

The changes in the POD activity of *T. urticae* in response to heat stress are shown in Figure 3. In comparison with controls, the POD activity was significantly enhanced at all temperatures (F = 322.98; *p* < 0.001) and durations (F = 63.41; *p* < 0.001) tested by two-way ANOVA, and there was a significant interaction among these treatment factors (F = 12.63; *p* < 0.001). The POD activity in *T. urticae*, recorded with only 391.69 U/mg protein, was lowest at 45 °C-6 h. The maximum value of POD activity was recorded as 1289.02 U/mg protein at 39 °C-4 h. The POD activity increased significantly (F = 121.74; *p* < 0.001) at 36–45 °C when the exposure time was 2 h and increased significantly (F = 559.327; *p* < 0.001) at 36, 39, and 42 °C when the exposure times were 4 and 6 h. No difference was found after exposure to 45 °C for 4 and 6 h.

### 3.4. Catalase (CAT) Activity

**Figure 4:**
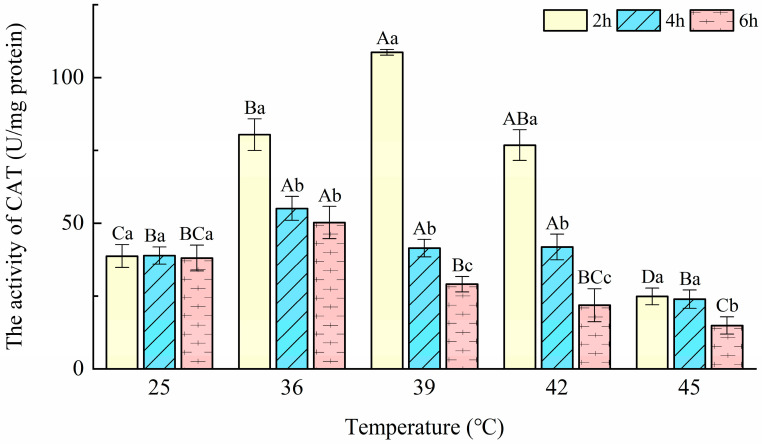
Effects of the activity of catalase (CAT) in *Tetranychus urticae* under heat stress.

The CAT activity of *T. urticae* under different heat stresses is presented in Figure 4. The two-way ANOVA showed that CAT activities were significantly affected by treatment temperature (F = 95.20; *p* < 0.001), duration (F = 124.85; *p* < 0.001), and their interaction (F = 18.31; *p* < 0.001). For three durations, the treatments at 45 °C significantly inhibited the CAT activity compared with controls (F_2h_ = 136.57, *p*_2h_ < 0.001; F_4h_ = 18.67, *p*_4h_ < 0.001; F_6h_ = 18.28, *p*_6h_ < 0.001). In a 2 h exposure at 39 °C, the CAT activity reached the maximum (recorded as 108.63 U/mg protein), 2.4 times higher than the control, and its minimum (14.67 U/mg protein) was showed at 45 °C-6 h. The CAT activity went up in the 2 h and 4 h treatments of 36, 39, and 42 °C compared with controls. When the exposure duration was extended to 6 h, it showed no significant difference at 42 °C compared with the control and was significantly higher than the controls at 36 °C and 39 °C (F = 18.28; *p* < 0.001).

### 3.5. Total Antioxidant Capacity (T-AOC)

**Figure 5:**
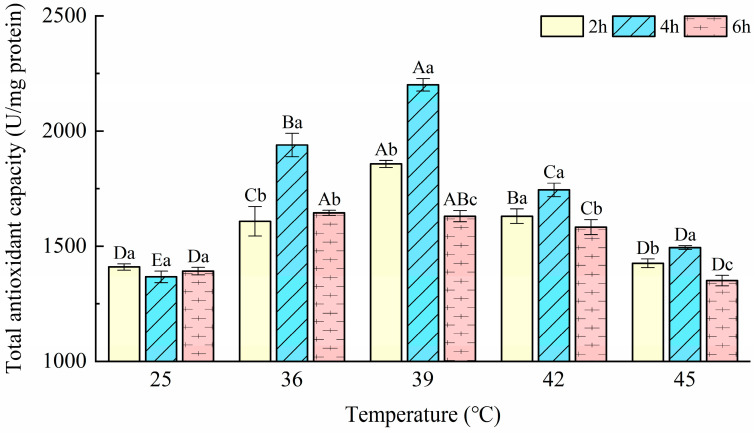
Effects of the activity of total antioxidant capacity (T-AOC) in *Tetranychus urticae* under heat stress.

As shown in Figure 5, T-AOC was significantly affected by treatment temperatures (F = 320.76; *p* < 0.001) and durations (F = 158.59; *p* < 0.001), and there was a significant interaction between temperature and duration (F = 35.00; *p* < 0.001). When the mites were treated at 36 °C, 39 °C, and 42 °C for three durations, T-AOC significantly increased compared with the controls (F_2h_ = 66.07, *p*_2h_ < 0.001; F_4h_ = 230.40, *p*_4h_ < 0.001; F_6h_ = 73.16, *p*_6h_ < 0.001) tested by the one-way ANOVA. The maximum (2200.97 U/mg protein) was present at 39 °C-4 h, and the minimum (1351.05 U/mg protein) was shown at 45 °C-6 h. When the exposure durations were 2 and 4 h, T-AOC showed the same trend that first increased and peaked at 39 °C, then decreased with rising temperature. After exposure to heat stress for 6 h, the highest value was attained at 36 °C, which was significantly higher than other treatments (F = 5.77; *p* = 0.037). When the temperature increased to 45 °C, T-AOCs at 2 h and 6 h were not significantly different from controls. However, T-AOC at 4 h was significantly higher than the control (F = 230.40; *p* < 0.001).

## 4. Discussion

Temperature is one of the most crucial environmental variables that can affect physiological changes in organisms [17]. Individual organisms will react to changes in temperature in a variety of ways, including positive or negative physiological and behavioral modifications [21,39,40]. To identify the oxidative stress and physiological responses of the *T. urticae* exposed to relatively high environmental temperature, the effects of temperature on antioxidant enzyme (SOD, POD, and CAT) activity and the changes in T-AOC and protein content were measured in this study. This is the first study on the influence of temperature on the antioxidant enzyme activity in *T. urticae*. The results showed that protective enzyme activities, total antioxidant capabilities, and protein content varied significantly compared with controls when subjecting *T. urticae* to different heat stresses, suggesting most ROS generated by heat stress were eliminated by these enzymes.

Superoxide dismutase (SOD) is among the most potent antioxidants known in nature and is an important constituent of cellular defense against oxidative stress [41]. In this study, SOD activity was induced significantly at the beginning of heat stress (36 and 39 °C) in comparison with the control. This means that SOD was induced by high temperatures to scavenge the superoxide anions in order to protect the *T. urticae* from heat stress. The results are consistent with the study in *Panonychus citri* (McGregor), whose SOD activity increased in response to high-temperature stress (32, 35, and 38 °C) [42]. Moreover, the SOD activity of the mite increased at the beginning of the stress and then decreased at 42–45 °C. This phenomenon may suggest that excessive ROS cause decreased activity of SOD or induce some other defense pathway. This is consistent with the findings of Yang et al. [42], where early-stage exposure to acute temperature changes resulted in oxidative stress regulated by antioxidant enzymes, but continued stress caused by acute temperature exposure resulted in decreased SOD activity. Previous research has demonstrated that negative feedback from an excess of substrate or oxidative modification can reduce enzyme activity [43]. The findings of both Jena et al. [44] in the silkworm *Antheraea mylitta* and Drury and Cui et al. [19] in *C. suppressalis* exposed to heat stress reported this phenomenon. These results suggest that the increased activity of SOD might be an adaptive response of *T. urticae* to overcome high-temperature-induced (≤42 °C) ROS toxicity.

Catalase (CAT) is the principal H_2_O_2_ scavenging enzyme, and CAT removes H_2_O_2_ only at high cellular concentrations, whereas it is inefficient for H_2_O_2_ removal at low concentrations [45]. In the present study, different increases in the activities of CAT were observed after exposing female adults of *T. urticae* to 36, 39, and 42 °C. The results indicated that heat stress might have elevated the H_2_O_2_ level in *T. urticae*, as a consequence of which CAT activities increased in concert to remove H_2_O_2_. These findings are similar to the study by Zhang et al. [12], who observed a significant increase in CAT activities in the *Neoseiulus cucumeris* (Oudeman) exposed to heat stress. Elevated CAT activities induced by heat stress were also observed in the oriental fruit fly [17]. The CAT activities at a treatment temperature of 45 °C were significantly lower than the controls. This may be due to that POD and other enzymatic and non-enzymatic substances removed most of the H_2_O_2_ at 45 °C, which made CAT remain at a low level. Another reason for decreased CAT activity may be the decrease in protein content. A similar case by Chen et al. [46] reported that CAT activities of male *Ophraella communa* LeSage adults decreased significantly at 44 °C compared with control (28 °C). In addition, in the study of Yang et al. [42], it was reported that the citrus red mite *P. citri* CAT activity was too low to detect. These results suggest that CAT participated in the response of *T. urticae* to short-term heat stress ≤42 °C.

Peroxidase (POD) also plays an important role in reducing H_2_O_2_ to H_2_O and O_2_. In this study, POD activity was significantly changed after being exposed to 36, 39, and 42 °C for 2, 4, and 6 h, in order to scavenge ROS to protect *T. urticae* from higher heat stress. *Propylaea japonica* (Thunberg), which is considered a successful natural enemy, has the same changes in POD activities to cope with heat stress [29]. POD activities at 45 °C were significantly decreased compared with the controls. The reasons for this situation may be the same as with CAT. From Figure 2 and Figure 3, we can see that POD activity is substantially higher than SOD activity. This indicated that, under heat stress, H_2_O_2_ was being produced directly by other processes in *T. urticae*. This was similar to the mechanism found in *Bactrocera dorsalis* (Hendel) [17]. According to the above content, POD plays a key role in decomposing the H_2_O_2_ produced in *T. urticae* after exposure to different temperatures in this study. The present results are in accordance with a study that reported that POD had an important role in the antioxidant response to heat stress in *O. communa* [46]. These results indicate that POD also provides protection for *T. urticae* under short-term heat stress at ≤42 °C.

The T-AOC assay has been widely utilized as a typical measure of total antioxidant capacity in organisms and as a tool to assess redox status [43,47]. In this study, the T-AOC of *T. urticae* exposed to heat stress increased significantly compared with the control except when stressed at 45 °C for 2 and 4 h, which both had an insignificant difference. This indicates that the T-AOC was sufficient to deal with oxidative stress after a brief impact by high temperature. This result is consistent with the study of Zhang et al. [12], where the T-AOC may be sufficient to deal with oxidative stress and free -radical formulation after exposing *N. cucumeris* to cold and heat shock. The value of the T-AOC at each treatment was higher than the sum of the values of activities of SOD, POD, and CAT. This may suggest that some non-enzymatic substances play a role in antioxidant stress. Salvucci et al. [48] reported that whiteflies accumulate the polyhydric alcohol and sorbitol when exposed to temperatures greater than about 30 °C. Łopieńska-biernat et al. [49] found that trehalose plays a key role in providing energy during the thermotolerance and starvation processes. In addition, heat-shock proteins and α-tocopherol can cooperate with antioxidant enzymes to deal with ROS damage [17]. The T-AOC decreased at a treatment temperature ≥42 °C, implying that some of the antioxidant mechanisms may be inhibited. It has been reported that the antioxidant system may be insufficient to eradicate the profuse creation of ROS under more severe stress circumstances [50]. Additionally, at the same temperature, with the extension of duration time, SOD, POD, and CAT activity and the T-AOC were changed, which suggested that various exposure times also effect SOD, POD, and CAT activity and the T-AOC. Li et al. found similar results in the study on the effects of short-term heat stress on the antioxidant enzymes of *N. barkeri* [51].

Moreover, we just investigated the influence of short-term heat stress on the lab population of *T. urticae* which was cultured at 25 °C. It is commonly believed that the tolerance of experimental populations cultured at 25 °C is lower than that of field populations. In addition, other physiological mechanisms and even molecular mechanisms also need to be explored when *T. urticae* is exposed to heat stress.

## 5. Conclusions

The short-term heat stress that disturbs the redox balance in *T. urticae* and leads to oxidative stress was investigated. To counter this stress, antioxidant enzymes providing antioxidant defense and protection that significantly enhanced SOD, POD, and CAT activities in response to heat stress are likely a defense mechanism against oxidative damage due to the accumulation of ROS. The higher levels of T-AOC in *T. urticae* serve as an important signal demonstrating that antioxidant enzymes are not always adequate to counteract the production of ROS induced by heat stresses. This means that *T. urticae* female adults have other antioxidant mechanisms in addition to antioxidant enzymes that can protect *T. urticae* female adults from the oxidative damage caused by heat stress. In summary, *T. urticae* can efficiently deal with ROS induced by thermal stress, which could partially explain how they survive at high temperatures. The findings here can provide a basis for further research on the molecular mechanisms of thermostability and a reference for the ecological adaptability of *T. urticae*.

## Data Availability

The data presented in the study are deposited in the Figshare repository, accession number https://figshare.com/s/418a4cc9141c75fa465d (accessed on 1 October 2022).
